# The Impacts of Local Anesthetics and Surgical Trauma on Immunity

**DOI:** 10.1155/vmi/3876353

**Published:** 2026-07-05

**Authors:** Maranata Milkias Kasto

**Affiliations:** ^1^ Department of Veterinary Medicine, Jinka University, P.O. Box 165, Jinka, Ethiopia

**Keywords:** immunity, local anesthetics, surgical trauma

## Abstract

Local anesthetics exert anti‐inflammatory effects through mechanisms that extend beyond mere sodium channel blockade. Surgical trauma is a potent iatrogenic trigger of systemic immune dysregulation. The stress response to surgery activates the hypothalamic–pituitary–adrenal (HPA) axis and the sympathetic nervous system. Local anesthetics modulate nearly every facet of the innate immune response and modulate adaptive immunity. Local anesthetics, via their inhibition of cytokine production and leukocyte activation, may consequently mitigate DAMP release by attenuating the amplification of tissue damage within the inflammatory cascade. Preexisting patient comorbidities significantly modulate the immune response to anesthesia and surgical intervention. Bupivacaine and ropivacaine exhibit differential effects on neutrophil extracellular trap (NET) formation. The immunomodulatory effects of local anesthetics exhibit heterogeneity dependent on the route of administration, dosing regimens, and frequency of application. Hypoxia serves as a critical modulator of anesthetic‐immune interactions. Surgical trauma frequently establishes a localized hypoxic microenvironment at the wound site. The interaction between anesthetic agents and anticancer therapies represents a burgeoning field of significant clinical relevance. Many chemotherapeutic agents exert their antitumor effects via immune‐mediated mechanisms, thereby suggesting that anesthetic‐induced immunosuppression could theoretically attenuate their efficacy. Surgical trauma and local anesthetics exert distinct yet interconnected influences on the immune system. Despite this observed potential, current evidence remains insufficient to warrant modifications in clinical practice. Addressing this knowledge gap necessitates large‐scale randomized controlled trials, comprehensive molecular‐level mechanistic investigations, and the development of validated immune biomarkers to inform perioperative anesthetic selection.

## 1. Introduction

Surgical trauma is a potent iatrogenic trigger of systemic immune dysregulation. The stress response to surgery activates the hypothalamic–pituitary–adrenal (HPA) axis and the sympathetic nervous system. This activation prompts the release of catecholamines, prostaglandins, cortisol, and a cascade of proinflammatory cytokines, which collectively suppress cell‐mediated immunity [1]. Natural killer (NK) cells, serving as the first line of innate antitumor defense, are particularly vulnerable; their cytotoxic activity is markedly reduced following surgical procedures, a phenomenon linked to an increased risk of metastatic recurrence [2]. Furthermore, cytotoxic T lymphocytes (CTLs), T helper (Th) cell subsets, macrophages, and neutrophils also exhibit functional impairment. This collective weakening creates a postoperative window of vulnerability, during which residual tumor cells may proliferate and disseminate [3].

However, the magnitude and character of this immunosuppression are not uniform, but rather depend critically on the type and duration of anesthesia administered. Volatile anesthetic agents such as isoflurane, sevoflurane, and desflurane have been shown to suppress NK cell cytotoxicity, induce T‐lymphocyte apoptosis, and upregulate hypoxia‐inducible factor‐1 alpha (HIF‐1alpha), thereby promoting an angiogenic and prometastatic microenvironment [1, 4]. Opioid analgesics further compound these effects by suppressing NK cell activity and expanding regulatory T‐cell populations [5]. In contrast, propofol‐based total intravenous anesthesia appears to better preserve immune function, while accumulating evidence suggests local anesthetics possess intrinsic, potentially protective, immunomodulatory properties [6, 7].

Local anesthetics exert anti‐inflammatory effects through mechanisms that extend beyond mere sodium channel blockade. Lidocaine and bupivacaine inhibit leukocyte adhesion to the vascular endothelium, reduce neutrophil migration and phagocytosis, attenuate the release of lysosomal enzymes and reactive oxygen species (ROS), and suppress the production of proinflammatory cytokines, including interleukin‐1 beta (IL‐1beta), interleukin‐8 (IL‐8), and tumor necrosis factor alpha (TNF‐alpha) [7, 8]. Cassuto et al. documented that the anti‐inflammatory potency of specific local anesthetics can, in certain contexts, surpass that of traditional nonsteroidal anti‐inflammatory drugs and corticosteroids, while simultaneously presenting a considerably more favorable adverse‐effect profile [7]. More recently, Wu et al. elucidated the signaling pathways mediated by lidocaine encompassing NF‐kappaB, TLR4/p38 MAPK, HIF‐1alpha, and TGF‐beta/Smad3, thereby revealing a complex, concentration‐dependent network of immunomodulatory effects that extend across both innate and adaptive immunity [8].

Beyond the classical NK and CTL axes, research has increasingly focused on more specific cellular targets. For instance, recent works demonstrate that local and regional anesthesia can influence macrophage polarization (M1/M2 balance), modulate neutrophil extracellular trap (NET) formation, and alter the function of myeloid‐derived suppressor cells (MDSCs) and dendritic cells [9, 10]. Furthermore, damage‐associated molecular patterns (DAMPs) such as HMGB1, HSP70, HSP90, and calreticulin are increasingly recognized as mediators of sterile perioperative inflammation, with emerging evidence suggesting that anesthetic agents may modulate DAMP release and signaling [11]. Therefore, understanding whether anesthesia exerts pro‐ or anti‐inflammatory effects and identifying the measurable biomarkers and key signaling pathways involved is essential for rationally selecting anesthetic strategies in oncological surgery [12].

The clinical implications are substantial. Given that up to 80% of cancer patients undergo surgery as part of their treatment, the perioperative period represents a critical window for immune preservation [13]. While retrospective analyses have suggested an association between regional anesthesia and local anesthetics and reduced cancer recurrence rates, prospective randomized trials have not consistently confirmed this benefit [14, 15].

Despite these clinical uncertainties, the experimental evidence for local anesthetics’ direct antiproliferative, antimigratory, and immune‐preserving effects is robust and continues to grow. This review aims to present a comprehensive, mechanistic synthesis of the interplay between local anesthetics and surgical trauma in modulating the immune response. Specifically, it examines their effects on innate and adaptive immune cell populations, the underlying molecular signaling pathways, the role of DAMPs and stress proteins, the dichotomous pro‐ and anti‐inflammatory facets of anesthetic action, and the clinical evidence supporting immune preservation.

## 2. The Surgical Stress Response and Immune Suppression

Surgical trauma elicits a complex neuroendocrine and immunological cascade. Tissue injury prompts the activation of the HPA axis and the sympathetic nervous system, culminating in the systemic dissemination of catecholamines, cortisol, prostaglandins, and proinflammatory cytokines [1, 4]. These mediators collectively attenuate cell‐mediated immunity, with the magnitude of suppression exhibiting a direct correlation with the severity of surgical insult [16].

A substantial impact on innate immunity is also observed. Circulating neutrophils exhibit demargination and a transient augmentation of phagocytic function in response to acute stress, attributable to catecholamine binding to beta‐2‐adrenergic receptors on the surface of leukocytes. However, this transient activation is succeeded by a more protracted phase of functional suppression. Simeonova et al. reported that both halothane general anesthesia and lidocaine epidural anesthesia in dogs resulted in concomitant elevations of adrenaline and cortisol, concurrent with discernible alterations in leukocyte distribution and phagocytic activity [17]. Cortisol directly inhibits lymphopoiesis and promotes accelerated lymphocyte apoptosis, thereby contributing to the postoperative lymphopenia typically observed in surgical patients [17, 18].

NK cells demonstrate significant susceptibility to perioperative stress. Their cytotoxic activity undergoes a precipitous decline in the immediate postoperative period, a phenomenon largely attributable to the synergistic effects of cortisol, prostaglandin E2, catecholamines, and transforming growth factor‐beta (TGF‐beta) secreted by perioperatively expanded MDSCs [2]. Angka et al. [19] elucidated the multifactorial nature of this NK cell dysfunction, which encompasses impaired granule exocytosis, diminished interferon‐gamma (IFN‐gamma) synthesis, and downregulation of activating receptors such as NKG2D and DNAM‐1. This culminates in a postoperative window of vulnerability wherein residual or circulating tumor cells may evade immune surveillance and subsequently establish metastatic foci.

Adaptive immunity exhibits analogous compromise. The ratio of Type 1 T helper (Th1) to Type 2 T helper (Th2) cells is skewed toward a Th2‐dominant profile, thereby diminishing cell‐mediated antitumor responses [4]. Concomitantly, CTL activity is attenuated, and regulatory T‐cell (Treg) populations proliferate, further exacerbating the immunosuppressive milieu [5]. Total lymphocyte counts undergo a reduction, predominantly due to a decrease in B lymphocytes, while CD8+ T cells may exhibit a transient increase attributable to catecholamine‐mediated mobilization from marginal lymphocyte pools [17].

Beyond the classical neuroendocrine axis, tissue damage induces the release of DAMPs, including HMGB1, HSP70, HSP90, S100 proteins, and calreticulin, thereby activating pattern recognition receptors such as Toll‐like receptors (TLRs) and the receptor for advanced glycation end‐products (RAGE) [11]. This sterile inflammatory response exacerbates immune dysregulation and contributes to postoperative complications, including infections, delayed wound healing, and potentially cancer recurrence [20].

## 3. Effects of Local Anesthetics on Immune Function

Local anesthetics exhibit a stark contrast to volatile agents and opioids, particularly concerning their influence on immune function. Lidocaine and bupivacaine have been shown to preserve or even enhance immune function through multiple independent mechanisms [6, 7].

Angjushev et al. demonstrated that patients undergoing mastectomy with either lidocaine infusion or pectoralis (PECS) I/II block with bupivacaine exhibited a significant increase in serum TNF‐alpha levels 24 h postsurgery, compared with patients receiving general anesthesia alone, suggesting enhanced immune activation. Furthermore, the PECS block cohort also demonstrated superior hemodynamic stability, reduced postoperative pain scores, lower opioid consumption, and a reduction in complications, including postoperative nausea and vomiting [4].

Chamaraux‐Tran and Piegeler [6] critically examined evidence indicating that lidocaine potentiates NK cell activity, directly suppresses cancer cell proliferation and migration, and attenuates the surgical stress response through its anti‐inflammatory properties. These effects are mediated by mechanisms independent of sodium channel blockade, encompassing the modulation of intracellular calcium signaling, G protein‐coupled receptors, and the cytoskeleton [7, 9].

Regional anesthetic modalities, such as paravertebral block, epidural anesthesia, and PECS blocks, confer further benefits by minimizing the required dosage of volatile anesthetics and opioids, thereby mitigating their immunosuppressive effects [5, 14]. Nevertheless, the clinical ramifications of regional anesthesia on long‐term oncological outcomes remain a domain of active inquiry, with definitive evidence from prospective randomized trials yet to be established [14, 15].

## 4. Cellular and Molecular Mechanisms of Local Anesthetic Immunomodulation

### 4.1. Effects on Innate Immune Cells

Local anesthetics modulate nearly every facet of the innate immune response. Cassuto et al. demonstrated that lidocaine and bupivacaine dose‐dependently inhibit leukocyte adhesion to vascular endothelium by interfering with integrin activation and leukocyte adhesion molecule‐1 expression, without compromising cell viability [7]. This inhibition occurs at clinically relevant plasma concentrations.

Leukocyte migration is likewise attenuated. Local anesthetics impair the spontaneous motility of neutrophils and diminish their chemotactic responsiveness to inflammatory signals, effects mechanistically attributed to the disruption of the actin cytoskeleton and the attenuation of chemoattractant release from activated leukocytes [7]. Phagocytosis is diminished in a dose‐dependent and reversible manner, primarily through the impairment of surface receptor expression and actomyosin filament activity [7, 21].

Bupivacaine and ropivacaine exhibit differential effects on NET formation. Kraus et al. demonstrated that bupivacaine induces an earlier onset of NETosis compared to ropivacaine, while both agents accelerate the cessation of neutrophil migration. These findings are clinically significant, as NETs have been implicated in tumor angiogenesis, immune evasion, and the promotion of metastasis. Sargarovschi et al. recently reported that lidocaine may reduce postoperative NET markers and other prometastatic factors, such as matrix metalloproteinase‐9 (MMP‐9) and vascular endothelial growth factor (VEGF) in cancer surgery patients [9].

Furthermore, macrophage polarization is also subject to modulation by local anesthetics. Wu et al. elucidated lidocaine’s effects on macrophage function via the NF‐kappaB, TLR4/p38 MAPK, and AMPK‐SOCS3 signaling pathways, demonstrating that lidocaine can shift macrophages toward an anti‐inflammatory phenotype under specific conditions [8]. The observed effects are concentration‐ and context‐dependent, and the equilibrium between pro‐ and anti‐inflammatory responses is modulated by the local microenvironment.

### 4.2. Effects on Adaptive Immune Cells

Local anesthetics have been demonstrated to modulate adaptive immunity. Specifically, lidocaine has been shown to inhibit the release of IL‐1β and IL‐8 from activated monocytes and macrophages, concurrently stimulating the secretion of the anti‐inflammatory interleukin‐1 receptor antagonist (IL‐1RA). This collective action suggests a reorientation toward immunoregulation. Furthermore, TNF‐α release is attenuated via the inhibition of Toll‐like receptor 4 (TLR4) and p38 mitogen‐activated protein kinase (MAPK) signaling pathways [7, 9].

Lidocaine’s influence on T‐lymphocyte subsets presents a complex profile. While lidocaine has been reported to suppress T‐cell proliferation at elevated concentrations, its administration at clinically relevant doses has been shown to preserve or even enhance the activity of cytotoxic T cells, thereby maintaining the Th1/Th2 balance. Moreover, a reduction in regulatory T cells (Tregs), which typically suppress antitumor immunity, has been observed following lidocaine treatment. This reduction represents a potential mechanism contributing to improved immune surveillance. B‐lymphocyte function and antibody production are similarly impacted. At high concentrations, lidocaine inhibits B‐lymphocyte proliferation and antibody secretion; however, the clinical significance of this particular effect within the perioperative period warrants further elucidation [9, 22].

### 4.3. Mode and Form of Anesthesia Administration and Its Effect

The immunomodulatory effects of local anesthetics exhibit heterogeneity dependent on the route of administration, dosing regimens, and frequency of application. Infiltrative anesthesia, involving direct injection of the agent into the surgical site, results in elevated local tissue concentrations capable of directly modulating immune cell function within the wound bed. This includes inhibition of neutrophil chemotaxis, reduced cytokine release, and suppression of nociceptive signaling [7, 9]. Conversely, regional techniques such as epidural, paravertebral, or PECS blocks primarily elicit systemic immune effects by attenuating the neuroendocrine stress response, decreasing catecholamine release, and lowering opioid requirements [4, 5]. Intravenous lidocaine infusion constitutes a third modality, producing plasma concentrations (1–5 mcg/mL) sufficient to exert anti‐inflammatory effects on circulating leukocytes without inducing neural blockade [6, 13].

A crucial distinction lies in the mode of administration: single‐dose versus repeated exposure. Acute, single‐dose exposure to local anesthetics during surgical procedures induces transient immunomodulation, which typically resolves within 24–48 h [7]. Conversely, repeated or continuous administration, exemplified by postoperative wound infusion catheters or prolonged epidural analgesia, may perpetuate immune suppression of neutrophil function beyond the operative period [21]. Bain et al. [23] have underscored that the inflammatory‐immune response to surgical injury can progress to a dysregulated state, necessitating careful calibration of repeated dosing of immunomodulatory agents to mitigate the exacerbation of this dysregulation.

### 4.4. Cytokine and Signaling Pathway Modulation

The immunomodulatory actions of local anesthetics are mediated by at least seven currently recognized distinct signaling pathways: NF‐kappaB, TLR4/p38 MAPK, voltage‐sensitive sodium channels, HIF‐1alpha, TGF‐beta/Smad3, AMPK‐SOCS3, and TBK1‐IRF7 [9]. This multiplicity of targets accounts for the broad spectrum of cellular effects observed. Within the arachidonic acid cascade, local anesthetics inhibit phospholipase A2 (PLA2) activity, diminish prostaglandin (PGE1, and PGE2) and thromboxane (TXB2) synthesis, and suppress leukotriene (LTB4) release from activated granulocytes [7]. These effects on eicosanoid production contribute to the antiedema and analgesic properties of local anesthetics.

Cytokine inhibition is dose‐dependent and encompasses IL‐1alpha, IL‐1beta, IL‐6, IL‐8, and TNF‐alpha [7, 9]. Clinically relevant concentrations of lidocaine infusion have been shown to significantly reduce systemic IL‐6 and IL‐8 levels following major surgery [24].

## 5. DAMPs, Heat Shock Proteins, and Sterile Inflammation

Surgical tissue injury instigates the release of a diverse array of endogenous DAMPs, which activate innate immune receptors and perpetuate sterile inflammation. Prominent DAMPs encompass high‐mobility group box 1 (HMGB1), heat shock proteins (HSP60, HSP70, and HSP90), S100A8/S100A9, calreticulin, adenosine triphosphate (ATP), and cell‐free DNA [11, 20]. These molecules engage with Toll‐like receptor 2 (TLR2), TLR4, TLR9, and the RAGE, thereby initiating NF‐kappaB‐dependent production of proinflammatory cytokines.

The magnitude of DAMP liberation is directly proportional to surgical invasiveness. Jacobs et al. demonstrated that mastectomy elicits a more substantial release of S100A8/A9 and S100A12 alarmins compared to breast‐conserving surgery, an observation associated with heightened intraoperative sympathetic activation and more pronounced postoperative immune suppression [25]. Furthermore, Leijte et al. reported significantly elevated peak HMGB1 levels in patients who developed postoperative infections following cytoreductive surgery, thereby suggesting DAMPs as prospective biomarkers of perioperative immune complications [20].

Local anesthetics, via their inhibition of cytokine production and leukocyte activation, may consequently mitigate DAMP release by attenuating the amplification of tissue damage within the inflammatory cascade. Heat shock proteins merit particular attention. HSP70 and HSP90 function as intracellular chaperones under physiological conditions; however, upon extracellular release following cellular stress or necrosis, they transform into immunostimulatory DAMPs [26].

## 6. Patient and Environmental Factors Modulating Anesthetic‐Immune Interactions

### 6.1. Anesthesia, Patient Condition, and Pathological Characteristics

Preexisting patient comorbidities significantly modulate the immune response to anesthesia and surgical intervention. For instance, hepatic dysfunction alters the metabolism and impairs the clearance of amide‐type local anesthetics (lidocaine, bupivacaine, and ropivacaine), leading to prolonged systemic exposure, an increased risk of toxicity, and potentially enhanced immunosuppressive effects on immune cells [27]. Similarly, pulmonary pathologies such as chronic obstructive pulmonary disease and asthma, characterized by baseline systemic inflammation and altered alveolar macrophage function, may potentiate the immunosuppressive effects of volatile anesthetics [28].

Cardiovascular disease introduces another layer of complexity. While local anesthetics such as lidocaine have been shown to reduce infarct size by attenuating leukocyte‐mediated tissue damage during myocardial ischemia and reperfusion injury [7], the hemodynamic instability associated with certain anesthetic techniques in patients with preexisting heart failure may amplify the stress response and exacerbate immune dysfunction. Furthermore, renal impairment delays the excretion of local anesthetic metabolites, thereby prolonging their anti‐inflammatory effects on circulating leukocytes [27].

Anemia, a prevalent comorbidity in cancer patients, independently contributes to perioperative immunosuppression. Bezu et al. demonstrated that anemia, alongside other preexisting factors such as malnutrition and prior transfusion, impairs immune effector cell function preoperatively [15]. The synergistic effect of anemia‐associated immune compromise and anesthetic‐induced immunosuppression may predispose patients to a heightened state of postoperative vulnerability.

### 6.2. Environmental Conditions: Hypoxia, Oxidative Stress, Anemia, and Metals

Hypoxia serves as a critical modulator of anesthetic‐immune interactions. Surgical trauma frequently establishes a localized hypoxic microenvironment at the wound site. Furthermore, volatile anesthetics, notably sevoflurane and isoflurane, upregulate HIF‐1alpha, thereby promoting angiogenesis, tumor cell survival, and immune evasion [1, 5]. Market et al. demonstrated tissue hypoxia as a key postoperative factor directly suppressing NK cell function and facilitating micrometastasis formation [2]. Conversely, lidocaine has been shown to inhibit HIF‐1alpha stabilization via its effects on mitochondrial function, potentially mitigating this hypoxia‐driven immunosuppression [9].

Oxidative stress is both a consequence and a driver of perioperative immune dysregulation. Surgical trauma generates ROS from activated neutrophils and damaged tissues, which can deplete antioxidant defenses and impair lymphocyte function [29]. This oxidative burden, often compounded by ischemia–reperfusion injury, drives mitochondrial dysfunction in immune cells. Wang et al. recently highlighted how this dysfunction alters cytokine production, phagocytic activity, and antigen presentation, thereby tipping the balance toward either excessive inflammation or postoperative immunosuppression [29]. Interestingly, local anesthetics have been shown to scavenge free radicals and inhibit superoxide anion production by neutrophils in a dose‐dependent manner, potentially mitigating some of these pro‐oxidative effects [7].

The presence of redox‐active metals, such as iron and copper, can further exacerbate oxidative tissue injury during ischemia–reperfusion; their levels are notably altered in hypoxic liver tissue [30]. While direct evidence linking metal ion disturbances to anesthetic‐induced immunosuppression is currently limited, this represents a critical gap in the literature that warrants investigation, particularly in patients with iron deficiency anemia or hemochromatosis undergoing surgery.

### 6.3. Combination of Anesthesia With Chemotherapy, Radiation Therapy, and Other Medications

The interaction between anesthetic agents and anticancer therapies represents a burgeoning field of significant clinical relevance. Many chemotherapeutic agents exert their antitumor effects via immune‐mediated mechanisms, thereby suggesting that anesthetic‐induced immunosuppression could theoretically attenuate their efficacy [15]. For instance, Tseng et al. [31] conducted a review elucidating the impact of perioperative anesthetic management on TNF‐alpha production, a cytokine pivotal for both chemotherapy efficacy and tumor progression. Furthermore, preclinical investigations have demonstrated that lidocaine can sensitize specific cancer cell lines to chemotherapeutic agents, thereby augmenting apoptosis via the modulation of intracellular signaling pathways [4, 9].

Beyond the realm of chemotherapy, radiation therapy induces immunogenic cell death, culminating in the release of DAMPs and tumor antigens that stimulate antitumor immunity—a phenomenon central to the abscopal effect [32]. The selection of anesthetic agents for procedures conducted prior to, concurrently with, or following radiotherapy may significantly modulate this immune activation. Similarly, locoregional therapies, such as irreversible electroporation and radiofrequency ablation, additionally remodel the tumor microenvironment, leading to enhanced antigen release and T‐cell activation [33]. However, the precise extent to which anesthetic agents amplify or attenuate these diverse immunological and microenvironmental effects remains incompletely elucidated.

Carnet Le Provost et al. conducted an extensive examination of local anesthetics in cancer therapy, demonstrating robust preclinical evidence for their direct antitumor properties. However, prospective randomized controlled trials have yet to corroborate these benefits in clinical settings, largely owing to the inherent challenges in mitigating confounding variables, such as concomitant chemotherapy and radiotherapy regimens [34].

### 6.4. Antigens and Immune Responses in Relation to Anesthesia

The direction and magnitude of the immune response during the perioperative period are profoundly influenced by the type and immunogenicity of encountered antigens. Surgical trauma, for instance, induces the release of a diverse repertoire of self‐antigens and DAMPs, such as HMGB1, HSP70, S100 proteins, and calreticulin, which activate pattern recognition receptors on dendritic cells and macrophages [11, 35]. The specific context of this antigen presentation, particularly the presence or absence of inflammatory signals, subsequently dictates the development of either immunological tolerance or immunity, thereby shaping the ensuing adaptive immune response. This mechanistic understanding bears significant implications for anesthetic selection. First, volatile anesthetics have been demonstrated to impair antigen presentation by dendritic cells and diminish the surface expression of major histocompatibility complex (MHC) Class II molecules [1, 5]. This effect potentially attenuates the adaptive immune response to tumor‐associated antigens liberated during surgical procedures. Second, local anesthetics may exert distinct modulatory effects on antigen‐presenting cell function. For instance, lidocaine has been reported to influence dendritic cell maturation and cytokine production in a concentration‐dependent manner [9], carrying potential implications for the processing and subsequent presentation of surgically liberated antigens to T lymphocytes. Fagundes et al.’s recent literature underscores that anesthetic agents can either suppress or enhance immune responses, with the resultant immunological outcome contingent upon drug type, dose, patient‐specific characteristics, and, crucially, the nature of the antigenic stimulus [36]. This intricate interplay encompassing anesthetic selection, antigen identity, and immune polarization remains largely underexplored in the extant literature.

## 7. NETosis and Macrophage Polarization

NET formation exhibits a dualistic function during the perioperative period: while NETs confer antimicrobial defense, excessive NETosis is implicated in the promotion of tumor angiogenesis, immune evasion, and metastasis [10]. Kraus et al. [37] reported that bupivacaine induces accelerated NETosis in human polymorphonuclear leukocytes compared with ropivacaine; furthermore, both agents were observed to expedite the cessation of neutrophil migration. These differential effects suggest that specific local anesthetics may differentially modulate NET formation, contingent upon their molecular structure and concentration.

Sargarovschi et al. [9] conducted a review examining the role of NETosis in hepatocellular carcinoma and its implications for anesthetic techniques. Their findings indicated that lidocaine may attenuate postoperative NET‐associated biomarkers (e.g., citrullinated histone H3, cell‐free DNA, and myeloperoxidase‐DNA complexes), as well as other prometastatic factors, including matrix MMP‐9 and VEGF. Consequently, lidocaine is posited as a potentially NET‐suppressive agent; however, the extant data remain preliminary.

Anesthetic agents significantly influence macrophage polarization, with local anesthetics capable of shifting the balance between proinflammatory (M1) and anti‐inflammatory (M2) macrophage phenotypes. For example, lidocaine inhibits M1 polarization by suppressing NF‐kappaB and TLR4/p38 MAPK signaling, and under certain conditions, enhances M2‐associated anti‐inflammatory cytokine production. Wu et al. further elucidated how lidocaine affects macrophage polarization through AMPK‐SOCS3 and TGF‐beta/Smad3 pathways, noting its context‐dependent effects that vary with concentration, exposure duration, and the local microenvironment [8, 9]. This modulation of macrophage polarization by anesthetic techniques may be crucial for postoperative pain control, as demonstrated by Celik et al., who showed that IL‐4‐induced M2 macrophages produce opioid peptides that sustain local analgesia [38].

## 8. Clinical Implications and Future Directions

The clinical significance of anesthetic‐induced immune modulation is particularly evident in oncological surgery. Retrospective investigations have indicated that regional anesthesia may be associated with reduced cancer recurrence rates, specifically in breast, prostate, and colorectal malignancies [5, 14]. Conversely, the inaugural large‐scale randomized controlled trial evaluating paravertebral regional anesthesia versus volatile anesthesia in breast cancer surgery reported no statistically significant disparity in recurrence‐free survival [14].

Based on the extant evidence, several promising avenues for future research warrant consideration. First, intravenous lidocaine infusion, employed as a perioperative adjuvant, merits further scrutiny, owing to its advantageous safety profile, opioid‐sparing attributes, and documented immunomodulatory capabilities [6, 13]. Second, the elucidation of dependable biomarkers—encompassing cytokine profiles, DAMP concentrations, and HSP expression—holds the potential to facilitate individualized anesthetic regimen selection for high‐risk patient cohorts [11, 39]. Third, the precise role of specific T‐cell subsets (e.g., Th17, Tfh, Th22, and Breg cells) and MDSCs in anesthetic‐induced immune modulation largely remains an uninvestigated domain, thereby constituting a notable lacuna in the existing literature.

The different effects of individual anesthetic agents on various immune cell populations under both inflammatory and anti‐inflammatory conditions are summarized in Table 1.

**TABLE 1:** Summary of individual anesthetics and their effects on immune cells under inflammatory and anti‐inflammatory conditions.

Anesthetic agent	Immune cell targets	Effects under inflammatory conditions	Effects under anti‐inflammatory/resting conditions	Experimental conditions	Key references
Lidocaine	Neutrophils	Inhibits adhesion, migration, phagocytosis, superoxide production, and NETosis	Reduces baseline chemotaxis; reversible inhibition without cytotoxicity	In vitro (human PMNs), in vivo (rat peritonitis, dog arthritis, human volunteers)	[7, 21, 37]
	Macrophages/monocytes	Suppresses TNF‐alpha, IL‐1beta, IL‐8 release; blocks NF‐kB and TLR4/p38 MAPK	Promotes IL‐1RA secretion; shifts toward an anti‐inflammatory phenotype at clinical concentrations	In vitro (human monocytes, murine macrophages), in vivo (rat lung injury)	[7, 9]
	NK cells	Preserves/enhances NK cytotoxicity; reverses opioid‐induced NK suppression	Increases baseline NK cell activity in vitro	In vitro (human NK cells), clinical (IV infusion during cancer surgery)	[6, 9]
	T lymphocytes	Inhibits T‐cell proliferation at high doses; preserves Th1/Th2 balance at low doses	Reduces Treg expansion; maintains CD8+ function	In vitro (human T cells), in vivo (animal models)	[9]
	Mast cells/DCs	Inhibits histamine release; modulates dendritic cell maturation	Reduces baseline degranulation	In vitro (human mast cells, murine DCs)	[7, 9]
	General cytokine profile	Reduces IL‐6, IL‐8, TNF‐alpha, HMGB1; blocks PGE2, TXB2, LTB4	Stimulates IL‐1RA secretion; preserves IL‐2 and IFN‐gamma	In vitro, in vivo (burn, lung injury), clinical (IV lidocaine infusion 1 mg/kg bolus + 1 mg/kg/h)	[7, 9, 31]

Bupivacaine	Neutrophils	Inhibits adhesion, migration, and induces earlier NETosis vs ropivacaine	Reduces baseline motility; prolongs clotting time via TXA2 inhibition	In vitro (human PMNs, platelet aggregation)	[7, 37]
	Monocytes	Inhibits IL‐1 release; suppresses TNF‐alpha‐induced IL‐8	Reduces spontaneous cytokine secretion	In vitro (human monocytes)	[7]
	NK cells/CTLs	Preserves NK cell activity in the PECS block; increases TNF‐alpha postsurgery	Maintains baseline immune function compared to GA alone	Clinical (PECS I/II block with 0.25% bupivacaine 0.2 mL/kg during mastectomy)	[4]
	Cancer cells (direct)	Induces apoptosis via caspase‐8 and caspase‐9 pathways; inhibits proliferation and migration	Cytotoxic at clinical concentrations; GSK‐3beta dependent in ovarian cancer	In vitro (SKOV‐3, PC‐3 cancer cell lines)	[40]
	Leukocytes (general)	Increases WBC and neutrophil counts 24 h postsurgery	Slight decrease in lymphocyte count	Clinical (PECS block, mastectomy patients)	[4]

Ropivacaine	Neutrophils	Weak or no inhibition of phagocytosis vs other LAs; causes earlier cessation of migration	No significant effect on baseline NETosis compared to bupivacaine	In vitro (human PMNs)	[7, 37]
	Keratinocytes	Suppresses proliferation and migration via PI3K/AKT/mTOR; increases IL‐6, TNF‐alpha	Reduces IL‐10 secretion; delays wound closure	In vitro (HaCaT cells), in vivo (rat wound model)	[41]
	Antibacterial	No direct antibacterial effect at clinical concentrations	Lacks antimicrobial activity (unlike lidocaine/bupivacaine)	In vitro (bacterial cultures)	[7]
	NK cells	No significant difference in NKCC vs fentanyl‐based analgesia	Preserves NK cell function comparable to opioid regimens	Clinical (ON‐Q wound infiltration 0.5%, laparoscopic colorectal surgery)	[42]

Abbreviations: CTL, cytotoxic T lymphocyte; DC, dendritic cell; EMT, epithelial–mesenchymal transition; HIF‐1alpha, hypoxia‐inducible factor 1‐alpha; LA, local anesthetic; MOR, mu‐opioid receptor; NETosis, neutrophil extracellular trap formation; NK, natural killer; PMN, polymorphonuclear leukocyte; RCT, randomized controlled trials; TLR, Toll‐like receptor.

## 9. Conclusion

Surgical trauma and local anesthetics exert distinct yet interconnected influences on the immune system. Surgical trauma induces a systemic neuroendocrine stress response that globally suppresses both innate and adaptive immunity. In contrast, local anesthetics, such as lidocaine and bupivacaine, exhibit intrinsic anti‐inflammatory and immunomodulatory properties. These properties encompass the preservation of NK cell activity, inhibition of excessive neutrophil activation, modulation of cytokine release, and attenuation of DAMP‐mediated sterile inflammation.

Consequently, the selection of an anesthetic technique may profoundly influence postoperative immune function, carrying significant implications for long‐term oncological outcomes. Despite this observed potential, current evidence remains insufficient to warrant modifications in clinical practice. Addressing this knowledge gap necessitates large‐scale randomized controlled trials, comprehensive molecular‐level mechanistic investigations, and the development of validated immune biomarkers to inform perioperative anesthetic selection.

## Funding

No funding was received for this manuscript.

## Conflicts of Interest

The author declares no conflicts of interest.

## Data Availability

Data sharing is not applicable to this article as no datasets were generated or analyzed during the current study.
